# ENOblock Does Not Inhibit the Activity of the Glycolytic Enzyme Enolase

**DOI:** 10.1371/journal.pone.0168739

**Published:** 2016-12-28

**Authors:** Nikunj Satani, Yu-Hsi Lin, Naima Hammoudi, Sudhir Raghavan, Dimitra K. Georgiou, Florian L. Muller

**Affiliations:** 1 Department of Neurology, McGovern Medical School, UTHealth, Houston, TX, United States of America; 2 Department of Cancer Systems Imaging, University of Texas MD Anderson Cancer Center, Houston, TX, United States of America; University of Nebraska Medical Center, UNITED STATES

## Abstract

Inhibition of glycolysis is of great potential for the treatment of cancer. However, inhibitors of glycolytic enzymes with favorable pharmacological profiles have not been forthcoming. Due to the nature of their active sites, most high-affinity transition-state analogue inhibitors of glycolysis enzymes are highly polar with poor cell permeability. A recent publication reported a novel, non-active site inhibitor of the glycolytic enzyme Enolase, termed ENOblock (N-[2-[2-2-aminoethoxy)ethoxy]ethyl]4-4-cyclohexylmethyl)amino]6-4-fluorophenyl)methyl]amino]1,3,5-triazin-2-yl]amino]benzeneacetamide). This would present a major advance, as this is heterocyclic and fully cell permeable molecule. Here, we present evidence that ENOblock does not inhibit Enolase enzymatic activity *in vitro* as measured by three different assays, including a novel ^31^P NMR based method which avoids complications associated with optical interferences in the UV range. Indeed, we note that due to strong UV absorbance, ENOblock interferes with the direct spectrophotometric detection of the product of Enolase, phosphoenolpyruvate. Unlike established Enolase inhibitors, ENOblock does not show selective toxicity to *ENO1*-deleted glioma cells in culture. While our data do not dispute the biological effects previously attributed to ENOblock, they indicate that such effects must be caused by mechanisms other than direct inhibition of Enolase enzymatic activity.

## Introduction

The inhibition of glycolytic/gluconeogenic enzymes is of interest in diverse area of medicine, including treatment of diabetes [1], the treatment of cancer [2] as well as the development of novel antimicrobials [3]. While high potency, transition state analogue active-site inhibitors of Enolase, and several other glycolytic enzymes have been described [4, 5], their utility is limited by poor cell permeability and otherwise poor pharmacological properties [6]. A recent report by Jung *et al*. described an altogether different glycolysis inhibitor: a heterocyclic, cell permeable inhibitor of Enolase that apparently binds outside the active site [7]. Jung *et al*. identified (N-2-2-2-aminoethoxy)ethoxy]ethyl]4-4-cyclohexylmethyl)amino]6-4-fluorophenyl)methyl]amino]1,3,5-triazin-2-yl]amino]benzeneacetamide hydrochloride) also known as AP-III-a4 as a compound selectively toxic to cancer cells under hypoxic conditions [7]. Further experiments led to the conclusion that AP-III-a4 exerts its effect by direct inhibition of the glycolytic enzyme Enolase with a reported IC_50_ of ~0.6 μM, hence it was dubbed ‘ENOblock’. This report piqued our interest as we had recently demonstrated that glycolytic enzymes can be targets of personalized cancer therapy if they belong to a family of paralogues, where one member is homozygously deleted in cancer. Specifically, we showed that passenger deletion of the 1p36 locus covering Enolase 1 (*ENO1*; αEnolase), leads to dramatic selective sensitization to ablation of Enolase 2 (*ENO2*; γEnolase) [8]. Enolase is an enzyme that catalyzes the second to last step in glycolysis, reversibly converting 2-phosphoglycerate (2-PGA) to phosphoenolpyruvate (PEP). While inhibitors of Enolase have been described, such as Phosphoacetohydroxamate [9, 10] and the natural antibiotic SF2312 [11–13], these compounds have very poor cell permeability and would make poor clinical candidates. As such, a cell permeable Enolase inhibitor would be of great utility as a potential clinical candidate for treating tumors with *ENO1*-homozygous deletion, as well as potentially other tumors that are heavily dependent on glycolysis.

Because of the potential utility of a cell permeable Enolase inhibitor for molecular targeted therapy of *ENO1*-deleted tumors, we systematically evaluated ENOblock as an inhibitor of Enolase *in vitro* and for selective killing of *ENO1*-deleted cancer cells. We find that ENOblock does not inhibit Enolase in *in-vitro* enzymatic assays and that it does not show selective toxicity towards *ENO1-*deleted cancer cells. We suggest that this discrepancy with conclusions in the paper by Jung *et al*. stems from the fact that the strong UV absorption of ENOblock interferes with the Enolase activity assay utilized in that publication [7], which relies on the rather weak UV absorption of PEP, the product of the Enolase reaction.

## Materials and Methods

### Enolase enzymatic activity

Native lysates of human cell lines were prepared using 20 mM Tris-HCl, 1 mM EDTA, and 1 mM β-mercaptoethanol at pH 7.4 and sonicated ten times for a period of 30s followed by cooling period of 30s, after which the lysates were cleared by centrifugation at 20,000g for 10 min. Enolase activity was measured using two different methods, either by 1) a fluorometric NADH-linked assay or 2) a direct spectrophotometric assay via formation of PEP. In the fluorometric assay, enolase activity was measured via NADH oxidation in a pyruvate kinase–lactate dehydrogenase coupled assay as previously described [8]. The assay is conducted in 10 mM KCl, 5 mM MgSO_4_, 100 mM triethanolamine at pH 7.4, with 400 μM NADH and 2 mM ADP. 2-Phosphoglycerate (2-PGA), pyruvate kinase (PK) and lactate dehydrogenase (LDH) are provided in excess, with conversion of 2-PGA to PEP by enolase being rate limiting. PEP (with ADP) is substrate of PK; pyruvate formed by this reaction is linked to NADH oxidation by LDH. Enolase activity is determined by measuring oxidation of NADH fluorescently by excitation at 340 nm and emission at 460 nm. The substrate concentration, if not otherwise indicated, was 2.5 mM 2-PGA. Fluorescence was measured using Omega Fluorescence Plate Reader (BMG Labtech). Alternatively, in a direct spectrophotometric assay, enolase activity was measured via the conversion of by 2-PGA to PEP by measuring absorption at 240 nm. The assay medium was the same, except that all the auxiliary reagents (PK/LDH, NADH, ADP) were omitted. Both assays were conducted in a 96-well plate format with the direct assay performed in UV-transmissible plates.

In addition, we repeated the direct spectrophotometric assay by utilizing the 50mM imidazole-HCl (pH 6.8), 2.0mM MgSO4 and 400 mM KCl buffer as described by Jung *et al* [7]. The reaction was initiated by adding 2.5 mM 2-PGA and optical density (OD) was measured at 240 nm using Omegastar Plate reader (BMG Labtech).

### Cell culture

The cell line D423-MG was kindly provided by D. Bigner [14]. The 1p36 homozygous deletion in D423 includes the genes from *CAMTA1 to SLC25A33*, which includes *ENO1*. Isogenic *ENO1* ectopically rescued lines were described previously (pCMV ENO1 5X, [8]). An *ENO1*-intact cell line (LN319) was used as a control for sensitivity to enolase inhibitors. Cells were routinely cultured in Dulbecco’s modified Eagle’s medium supplemented with 10% fetal bovine serum.

### Proliferation assays

Cell Proliferation was determined by crystal violet staining. We used D423 cell line (*ENO1*-deleted), D423 ENO1 (overexpressing *ENO1*) and LN319 (control cell line). Briefly, glioma cells were seeded in 96-well plates and treated with different concentrations of ENOblock or SF2312 for 7 days. Cells were then washed with PBS, fixed with 10% formalin and stained with 0.05% crystal violet. Washed and dried plates were dye-extracted using 10% acetic acid, and absorbance was measured at 595 nm using Omegastar Plate Reader (BMG Labtech). To test drug’s efficacy on cell proliferation under hypoxia, 1 x 10^4^ cells were plated in 96-well plates, treated with ENOblock and SF2312 and incubated for 3 days in a hypoxia station (Don Whitley Scientific, Shipley, UK) set at 0.1% O_2_ and 5% CO_2_. Crystal violet staining was then performed as described above.

### NMR determination of enolase activity

Reactions were conducted in a standard quartz NMR tube in 500 uL of Imidazole enolase activity buffer with 2 mM of 2-PGA added as a substrate. Phosphorus spectra (1300 transients) were acquired at the M.D. Anderson NMR Core in the proton decoupled mode on a 300 MHZ Bruker instrument.

### Enolase inhibitors

SF2312 was prepared by the M.D. Anderson Chemistry core using a slightly modified procedure as previously described [15]. A complete description of the SF2312 and its role as an Enolase inhibitor has been described by our group previously [12]. AP-III-a4 (ENOblock) was purchased from Sellekchem (Catalog No.S7443, CAS# 1177827-73-4). The identity of this product with the published material was confirmed by high resolution mass spec conducted at the Baylor College of Medicine Mass Spec Core. calculated [M + H]^+^ C_13_H_43_FN_8_O_3_ 595.3515, observed [M + H] ^+^ 595.3543; versus 595.3538 observed by Jung *et al*.

### Recombinant enolase proteins

Recombinant ENO1 and ENO2 proteins used in our experiments were generated by the Core for Biomolecular Structure and Function. Purity of both recombinant ENO1 and ENO2 proteins was verified using Ponceau and Coomasie Blue staining. In addition, western blots were carried out to verify the purity of recombinant ENO1 and ENO2 isoforms using following antibodies: anti-ENO1 (1:1000 dilution, Abcam ab155102), anti-ENO2 (1:1000 dilution, Dako M087301-2) and Pan-Enolase antibody (1:1000 dilution, Abcam ab189891).

### Statistical analysis

Generalized linear regression model was used to evaluate the inhibitory effect of ENOblock and SF2312 in NMR determination of Enolase activity over time. Generalized estimating equation method was used to measure correlation within samples at different time-points. Statistical analysis for this linear regression model was performed using SAS 9.4. Repeated measures one-way ANOVA with Bonferroni correction was used to estimate the inhibitory effects of a dose response with ENOblock and SF2312 on different cell lines. To evaluate differences in cell proliferation between normoxic and hypoxic conditions, multiple t-tests were used at specified dose. Data with p-values < 0.05 were considered significantly different. All ANOVA and t-tests were performed using GraphPad Prism 7.

## Results and Discussion

**Fig 1:**
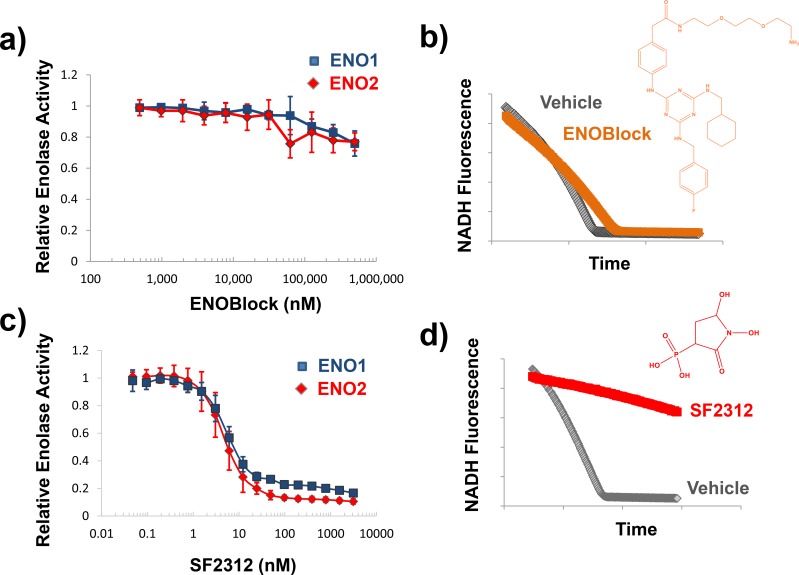
Fluorometric NADH-linked assay for measuring Enolase activity. The effect on ENOblock **(a, b)** and SF2312 **(b, d)** on Enolase activity in lysates from the D423 cell line expressing ENO2 (red diamonds) or ENO1 (blue squares) was determined using the NADH-linked assay. Panel **b** shows a representative trace of NADH fluorescence over time of vehicle control (gray symbols) and 100 μM ENOblock (orange symbols), while Panel **d** shows traces of vehicle control (gray symbols), with 50 nM SF2312 (red symbols). Panel **a and c** shows enolase activity normalized to vehicle control and expressed as a function of inhibitor concentration. Each data point represents mean of N = 6 (**a)** and N = 4 (**c**) ± S.D.

**Fig 2:**
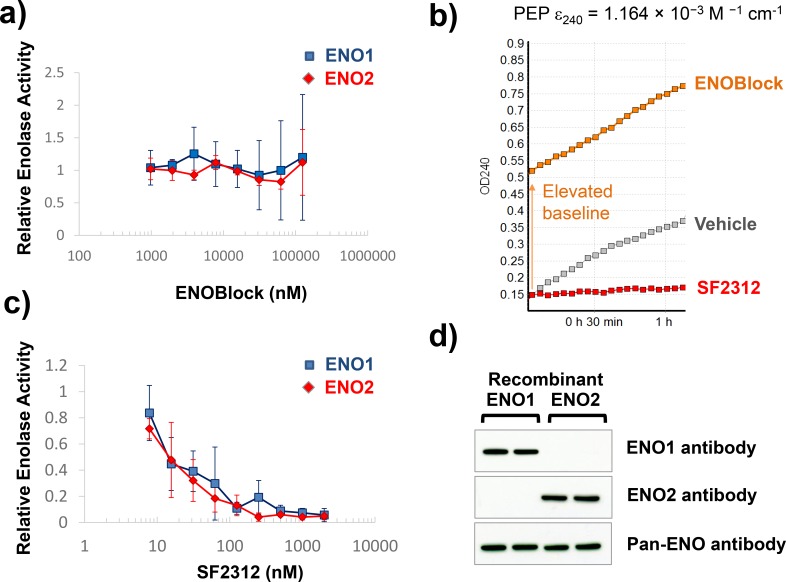
Effect of ENOblock on Enolase activity using spectrophotometric detection of PEP. The effect on ENOblock **(a)** and SF2312 **(c)** on Enolase activity in purified recombinant ENO1 and ENO2 was determined using the direct PEP-detection assay. Panels **a** and **c** show enolase activity normalized to vehicle control and expressed as a function of inhibitor concentration. Each data point represents mean of N = 4 ± S.D. Panel **b** shows a representative traces of absorption at 240 nm over time of vehicle control; 62,000 nM ENOblock and 100 nM SF2312. Note the increased baseline with ENOblock. Panel **d** shows western blots for recombinant proteins blotted with ENO 1, ENO2 and pan-ENO antibodies.

**Fig 3:**
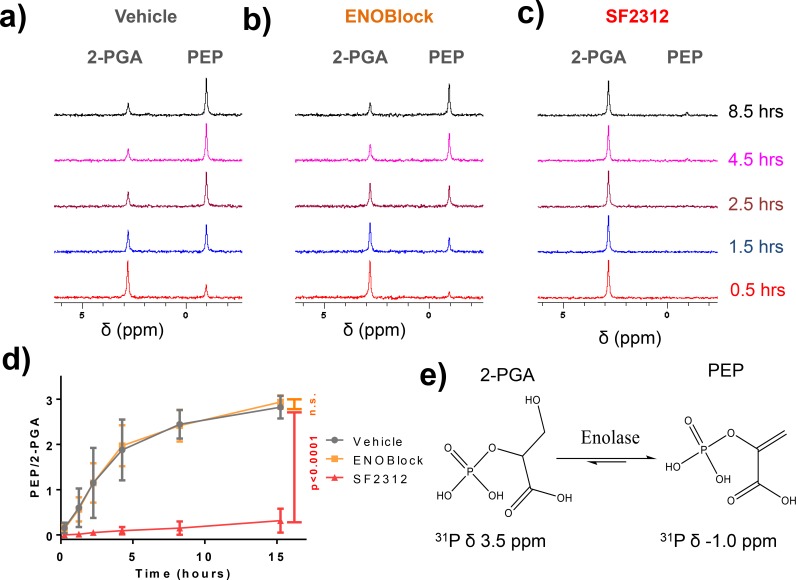
ENOblock does not inhibit the conversion of 2-PGA to PEP as measured by ^31^P NMR Experimental conditions were the same as for the experiments presented in Fig 2, except that 10% D_2_O was added for signal lock and the experiment was performed at room temperature. ^31^P NMR scanning was performed with 1300 transients, totaling 1 hour of measurement with the first time point being taken as half the time of measurement (0.5hr). (**a)** The 2-PGA peak (3.5 ppm, **e**) was stable for >24hrs in the absence of enzyme, but rapidly decreased with a concomitant increase in the PEP peak (-1ppm, **e**) in the presence of 10 nM recombinant hENO2. (**b)** Pre-incubation with 500 μM ENOblock did not slow the conversion of 2-PGA to PEP, but 20 μM SF2312 eliminated it Panel (**c**). (**d**)The ratio of integrals of 2-PGA and PEP as a function of time are shown for vehicle (grey), 500 μM ENOblock (orange), and 20 μM SF2312 (red). Each data point represents mean of N = 4 ± S.D. Differences between vehicle and ENOblock was not significant (n.s. = not significant; p = 0.69); while that between vehicle and SF2312 was highly significant (p<0.0001; Generalized linear regression model). Panel (**e**) shows the structure of 2-PGA and PEP with the published chemical shifts in the ^31^P spectrum.

The effect of ENOblock and other inhibitors on Enolase activity *in vitro* was determined using an NADH-coupled assay utilizing lysates of cancer cell lines overexpressing ENO1 and ENO2 (D423-ENO1 and D423-ENO2 respectively). In this assay, the formation of PEP (from 2-PGA supplemented in the assay) is linked to NADH oxidation via Lactate dehydrogenase and Pyruvate Kinase [16]. We find that concentrations up to 500 μM ENOblock fail to inhibit the oxidation of NADH, i.e. do not inhibit Enolase activity (Fig 1A and 1B). In contrast, as little as 50 nM of the active site inhibitor, SF2312, decreased the rate of NADH oxidation (Enolase activity) by >80% (Fig 1C and 1D). We also measured the effect of ENOblock on enolase activity using a direct assay, where the appearance of PEP was monitored by UV absorption of its double bond (240 nm) [17]. Indeed, in the work of Jung *et al*., the only data directly supporting inhibition of Enolase activity by ENOblock was an end-point assay detecting PEP at 240 nm (before and after 10 minutes following addition of 2-PGA) using recombinant enzyme [7]. We aimed to reproduce their exact methodology, however, rather than performing an end-point measurement, we performed this measurement kinetically. During the course of these experiments we found that ENOblock dramatically raises the baseline UV absorption, before substrate is added (Fig 2). This is not surprising as conjugated aromatic systems such as those in ENOblock are well known to strongly absorb in that region of the UV spectrum. We found that measurements had very high variability, especially at higher concentration of inhibitor, and it proved difficult to reach a firm conclusion as to whether the ENOblock yielded genuine enzymatic activity inhibition (Fig 2A and 2B). At the same time, the active site Enolase inhibitor SF2312 inhibited PEP formation with similar potency as determined in the NADH coupled assay (Fig 2C). We verified the purity of our recombinant proteins to exclude the possibility of any impurities causing this variability (Fig 2D, Figure A in S2 Fig). To avoid this complication of the spectrophotometric assay, we developed a novel enolase activity assay, where the conversion of 2-PGA to PEP was measured by ^31^P NMR utilizing identical conditions but performing the experiment in a standard quartz NMR tube. 2-PGA is evident as a peak at ^31^P 3.5 ppm [18]; upon addition of 10 nM recombinant Enolase 2, the 2-PGA peak gradually decreased with concomitant appearance of a peak at -1.0 ppm (Fig 3A), consistent with literature values for PEP [18]. Addition of 500uM of ENOblock with recombinant enolase failed to inhibit the appearance of ^31^P NMR PEP peak (p = 0.69) (Fig 3B). On the other hand, 20 μM SF2312 with recombinant Enolase fully abrogated the appearance of the PEP peak (p<0.0001) (Fig 3C), providing direct evidence for inhibition of Enolase enzymatic activity.

Taken together these data, from the three different assays, all unequivocally demonstrate Enolase inhibition by SF2312, providing a “positive control” of how an Enolase inhibitor is expected to function. ENOblock fails to inhibit enolase activity as measured by the fluorometric NADH linked assay, while providing highly variable results with the spectroscopic PEP detection assay. However, ^31^P NMR clearly shows absence of inhibition by ENOBlock, and thus the most parsimonious explanation is that ENOblock interferes with the UV absorption assay. This interference of ENOblock with the spectrophotometric detection of PEP would not have been obvious using an endpoint method assay as described by Jung et al [7]. We performed these experiments with ENOblock purchased from Sellekchem; the same experiments were also repeated with ENOblock shared by Jung et al., with the same results (data not shown). Taking these findings into consideration, we conclude that the spectroscopic assay is not an appropriate assay to measure inhibition of Enolase activity by ENOBlock.

**Fig 4:**
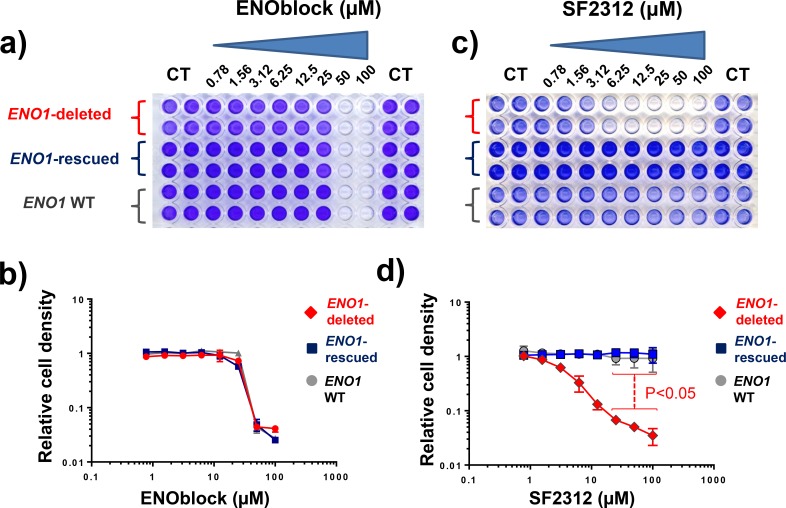
Non-selective toxicity of ENOBlock to ENO1-deleted glioma cells. A representative plate of cancer cells treated with ENOblock is shown in panel **a**, with quantification shown in panel **b** A plate treated with SF2312 is shown in panel **c**, with quantification shown in panel **d**. Cell were treated for 7 days. **(b, d)** D423 *ENO1*-deleted (red diamonds), D423 *ENO1*-rescued (blue squares) and LN319 *ENO1* WT (grey circles) were treated with the indicated doses of ENOblock in panel **b** (N = 4 ± S.D) or SF2312 in panel **d** (N = 4 ± S.D). Cell density was quantified by crystal violet and expressed relative to vehicle control as a function of inhibitor concentration. At high concentrations, SF2312 selectively killed D423 *ENO1*-deleted cells as compared to D423 *ENO1*-rescued cells (p<0.05, Repeated Measures one-way ANOVA with Bonferroni correction). ENOblock failed to show such selectivity regardless of dose.

Previous work from our group demonstrated that glioma cells with deletion of ENO1 show >90% decrease in total Enolase activity and dramatic selective sensitivity to the pan-Enolase inhibitor, Phosphonoacetohydroxamate [8]. To determine whether or not ENOBlock might exhibit selective toxicity towards *ENO1*-deleted glioma cells we tested ENOBlock on the same cell lines. Treatment with ENOblock over the course of 7 days, showed near equal toxicity to D423 *ENO1-*deleted glioma cells and an isogenic rescued cell line ectopically re-expressing ENO1, as well as *ENO1*-intact glioma cell lines (LN319). ENOblock at concentrations >25 μM eradicated glioma cells regardless of ENO1 status (Fig 4A and 4B). As a positive control, we determined the effects of the active site Enolase inhibitor SF2312 on D423 *ENO1*-deleted, an isogenic rescued cell line ectopically over-expressing ENO1, and *ENO1*-intact glioma cells (LN319). SF2312 showed strong selective toxicity towards *ENO1*-deleted glioma cells with *ENO1*-intact glioma cells being minimally affected at up to 100 μM inhibitor (p<0.05) (Fig 4C and 4D). We also performed short term, 3-day, treatments to determine the effect of hypoxia on toxicity of ENOblock and SF2312. Consistent with published work [7], hypoxia increased the toxicity of ENOblock towards all glioma cell lines, regardless of *ENO1*-deletion status (Figure A and B in S1 Fig). The selective toxicity of SF2312 towards *ENO1*-deleted glioma cells was evident at normoxia, but the effect was dramatically potentiated under hypoxia (Figure C and D in S1 Fig). At concentrations >12.5 μM SF2312 completely eradicated *ENO1*-deleted glioma cells under hypoxia. Under normoxia, similar concentrations inhibited proliferation resulting in lower cell density, but not complete eradication. This increased sensitivity of *ENO1*-deleted cells to the enolase inhibitor SF2312 under hypoxia is fully expected, given that energy generation by the mitochondrial respiratory chain is expected to be impaired under these conditions, resulting in increased reliance on glycolysis.

In conclusion, our data quite unequivocally indicate that ENOblock does not inhibit the enzymatic activity of Enolase. That said, in at least one aspect, sensitization to hypoxia, our data agree with the biological effects of ENOblock reported by Jung *et al*. Indeed, our results do not challenge the validity of the many interesting biological effects of ENOblock that Jung *et al* reported. However, identifying the correct mechanism will likely prove challenging. While our data indicate that ENOBlock does not inhibit the enzymatic activity of Enolase, they do not dispute that ENOblock may bind to Enolase (Figure 2a in [7]). However, no additional data, such as X-ray structures, Cellular thermal shift assays or mutational analysis, which would indicate the specific binding site of ENOBlock on Enolase were presented in Jung *et al*. Furthermore, ENOblock appears to bind to several additional proteins (Figure 2a in [7]) besides Enolase. Thus, while we can conclude that ENOBlock acts through a mechanism other than direct inhibition of the enzymatic activity of Enolase, this mechanism remains unknown and determining how ENOBlock exerts its reported biological effects is not immediately clear and will likely require further extensive experimentation.

## Supporting Information

S1 Fig Effect of Hypoxia on sensitivity to ENOblock and SF2312. D423 *ENO1*-deleted (red diamonds), D423 *ENO1*-rescued (blue squares) and LN319 *ENO1*-WT (grey circles) glioma cells were treated with indicated ENOblock doses (Panel **a** and **b**) or SF2312 (Panel **c** and **d**) and incubated either at 21% O_2_ indicated as Normoxia or 0.1% O_2_ indicated as Hypoxia for 3 days. Cell density was quantified by crystal violet and expressed relative to vehicle control as a function of inhibitor concentration (Panels **b a**nd **d**). Each data point represents mean of N = 4 ± S.D. Differences between hypoxic and normoxic conditions for *ENO1*-deleted glioma cells significant to at least p<0.01 are indicated (unpaired t-test with Bonferroni correction).
(TIF)

S2 Fig Purity analysis of recombinant ENO1 and ENO2. Panel **a** shows purity of recombinant ENO1 and ENO2 proteins by Ponceau staining and Coomasie staining. Panel **b** shows uncropped western blots from Fig 2 (Red rectangle indicates the blots used in Fig 2 for recombinant ENO1 and ENO2 proteins blotted with their respective antibodies (ENO1 antibody, 1:1000, Abcam ab155102; ENO2 antibody, 1:1000, Dako M087301-2 and Pan-Enolase antibody, 1:1000, Abcam ab189891).
(TIF)

## References

[1] Erion DM, Lapworth A, Amor PA, Bai G, Vera NB, Clark RW, et al The hepatoselective glucokinase activator PF-04991532 ameliorates hyperglycemia without causing hepatic steatosis in diabetic rats. PLoS One. 2014;9(5):e97139 10.1371/journal.pone.0097139 24858947 PMC4032240

[2] Locasale JW, Vander Heiden MG, Cantley LC. Rewiring of glycolysis in cancer cell metabolism. Cell Cycle. 2010;9(21):4253 10.4161/cc.9.21.13925 21045562

[3] de ASNMV, Gomes Dias SM, Mello LV, da Silva Giotto MT, Gavalda S, Blonski C, et al Structural flexibility in Trypanosoma brucei enolase revealed by X-ray crystallography and molecular dynamics. FEBS J. 2007;274(19):5077–89. 10.1111/j.1742-4658.2007.06027.x 17822439

[4] Alahuhta M, Wierenga RK. Atomic resolution crystallography of a complex of triosephosphate isomerase with a reaction-intermediate analog: new insight in the proton transfer reaction mechanism. Proteins. 2010;78(8):1878–88. 10.1002/prot.22701 20235230

[5] Dax C, Duffieux F, Chabot N, Coincon M, Sygusch J, Michels PA, et al Selective irreversible inhibition of fructose 1,6-bisphosphate aldolase from Trypanosoma brucei. J Med Chem. 2006;49(5):1499–502. 10.1021/jm050237b 16509566

[6] Pradere U, Garnier-Amblard EC, Coats SJ, Amblard F, Schinazi RF. Synthesis of nucleoside phosphate and phosphonate prodrugs. Chem Rev. 2014;114(18):9154–218. 10.1021/cr5002035 25144792 PMC4173794

[7] Jung DW, Kim WH, Park SH, Lee J, Kim J, Su D, et al A unique small molecule inhibitor of enolase clarifies its role in fundamental biological processes. ACS Chem Biol. 2013;8(6):1271–82. 10.1021/cb300687k 23547795

[8] Muller FL, Colla S, Aquilanti E, Manzo VE, Genovese G, Lee J, et al Passenger deletions generate therapeutic vulnerabilities in cancer. Nature. 2012;488(7411):337–42. 10.1038/nature11331 22895339 PMC3712624

[9] Poyner RR, Reed GH. Structure of the bis divalent cation complex with phosphonoacetohydroxamate at the active site of enolase. Biochemistry. 1992;31(31):7166–73. 1322695 10.1021/bi00146a020

[10] Anderson VE, Weiss PM, Cleland WW. Reaction intermediate analogues for enolase. Biochemistry. 1984;23(12):2779–86. 6380574 10.1021/bi00307a038

[11] Watanabe H, Yoshida J, Tanaka E, Ito M, Miyadoh S, Shomura T. Studies on a new phosphonic acid antibiotic, SF-2312. Sci Rep Meiji Seika Kaisha. 1986;25:12–17.

[12] Leonard PG, Satani N, Maxwell D, Lin YH, Hammoudi N, Peng Z, et al SF2312 is a natural phosphonate inhibitor of enolase. Nat Chem Biol. 2016. 10.1038/nchembio.2195 PMC5110371 27723749

[13] Muller F, Maxwell DS, Bornmann WG, Lin YH, Prasad BAB, Peng Z, et al. Enolase inhibitors and methods of treatment therewith. US patent WO2016145113 A1. 2016.

[14] Duncan CG, Killela PJ, Payne CA, Lampson B, Chen WC, Liu J, et al Integrated genomic analyses identify ERRFI1 and TACC3 as glioblastoma-targeted genes. Oncotarget. 2010;1(4):265–77. 10.18632/oncotarget.137 21113414 PMC2992381

[15] Hanaya and Itoh, An effective synthesis of antibiotic SF-2312 (3-dihydroxyphosphoryl-1,5-dihydroxy-2-pyrrolidone, Heterocycles, 82(2):1675–1683, 2011.

[16] Muller F, Aquilanti E, DePinho R. In vitro enzymatic activity assay for ENOLASE in mammalian cells in culture. 2012.

[17] Pancholi V, Fischetti VA. alpha-enolase, a novel strong plasmin(ogen) binding protein on the surface of pathogenic streptococci. J Biol Chem. 1998;273(23):14503–15. 9603964 10.1074/jbc.273.23.14503

[18] Thompson J, Torchia DA. Use of 31P nuclear magnetic resonance spectroscopy and 14C fluorography in studies of glycolysis and regulation of pyruvate kinase in Streptococcus lactis. J Bacteriol. 1984;158(3):791–800. 6427193 10.1128/jb.158.3.791-800.1984 PMC215511

