# Supplementary figures and images for: ENOblock Does Not Inhibit the Activity of the Glycolytic Enzyme Enolase
Source: PLoS One. 2016 Dec 28;11(12):e0168739. doi: 10.1371/journal.pone.0168739 (PMC5193436; doi:10.1371/journal.pone.0168739)

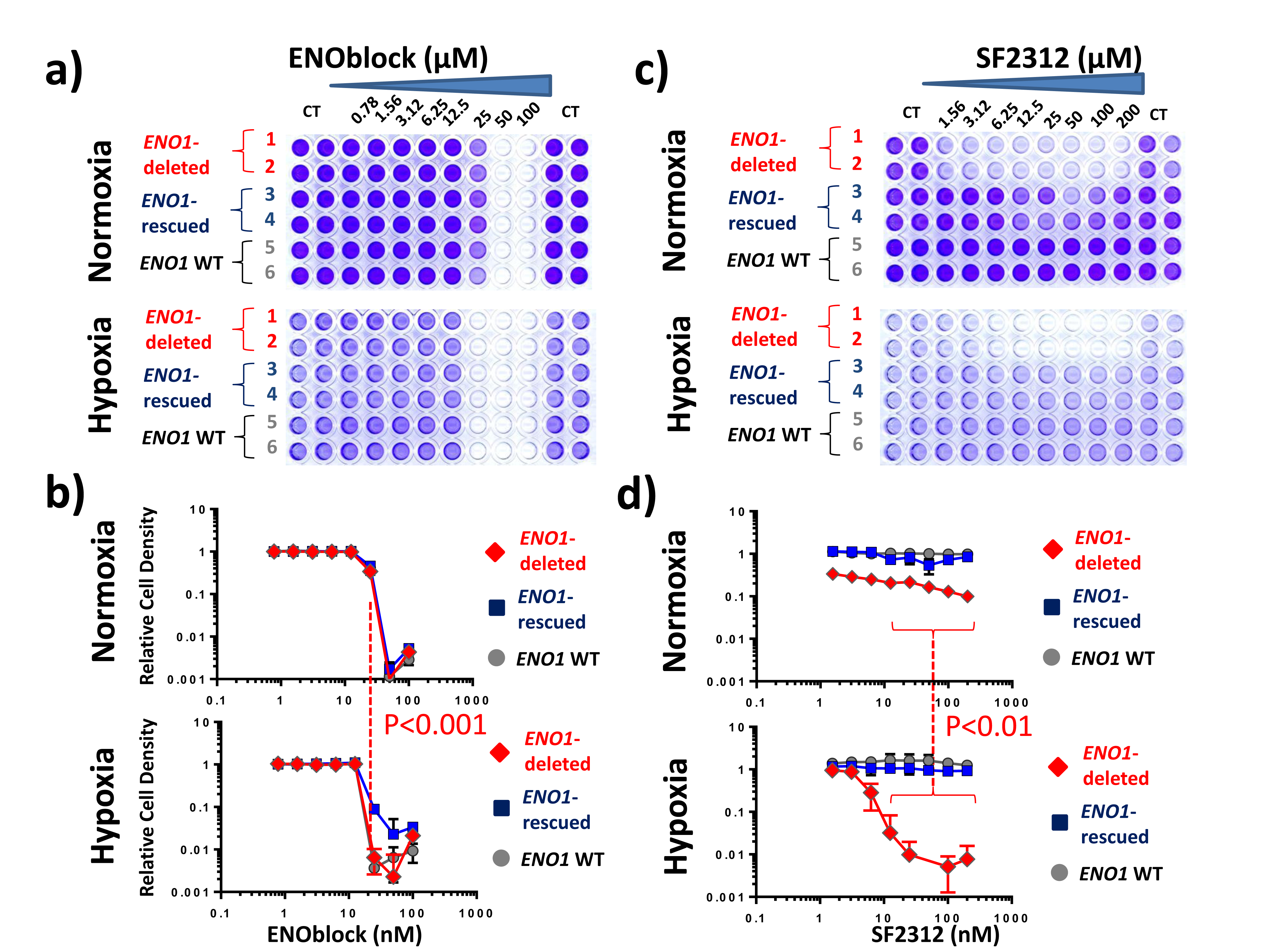

Supplement: S1 Fig — D423 ENO1-deleted (red diamonds), D423 ENO1-rescued (blue squares) and LN319 ENO1-WT (grey circles) glioma cells were treated with indicated ENOblock doses (Panel a and b) or SF2312 (Panel c and d) and incubated either at 21% O2 indicated as Normoxia or 0.1% O2 indicated as Hypoxia for 3 days. Cell density was quantified by crystal violet and expressed relative to vehicle control as a function of inhibitor concentration (Panels b and d). Each data point represents mean of N = 4 ± S.D. Differences between hypoxic and normoxic conditions for ENO1-deleted glioma cells significant to at least p<0.01 are indicated (unpaired t-test with Bonferroni correction). (TIF) [file pone.0168739.s001.tif]

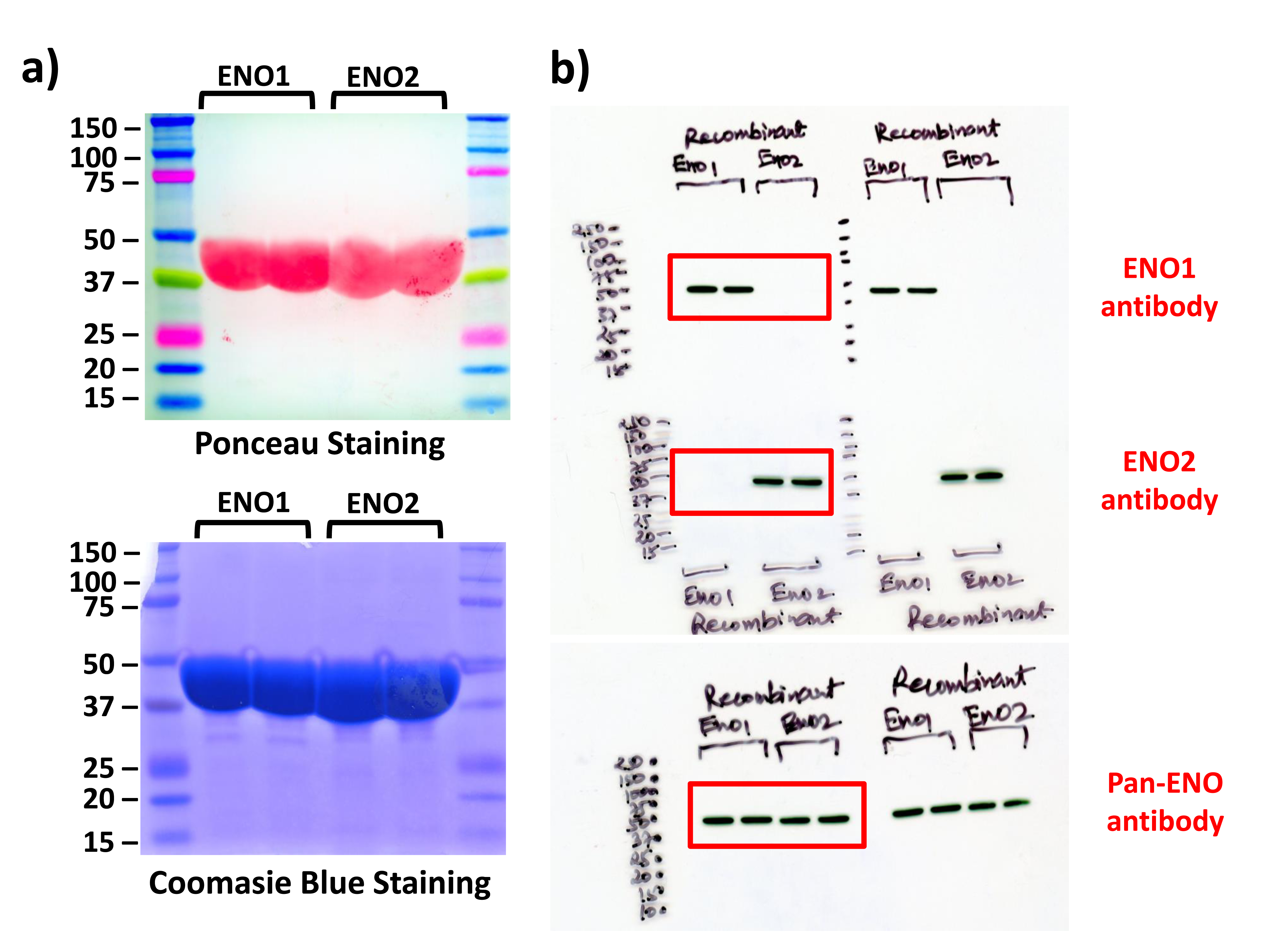

Supplement: S2 Fig — Panel a shows purity of recombinant ENO1 and ENO2 proteins by Ponceau staining and Coomasie staining. Panel b shows uncropped western blots from Fig 2 (Red rectangle indicates the blots used in Fig 2 for recombinant ENO1 and ENO2 proteins blotted with their respective antibodies (ENO1 antibody, 1:1000, Abcam ab155102; ENO2 antibody, 1:1000, Dako M087301-2 and Pan-Enolase antibody, 1:1000, Abcam ab189891). (TIF) [file pone.0168739.s002.tif]
